# Highly Effective Broad Spectrum Chimeric Larvicide That Targets Vector Mosquitoes Using a Lipophilic Protein

**DOI:** 10.1038/s41598-017-11717-9

**Published:** 2017-09-12

**Authors:** Dennis K. Bideshi, Hyun-Woo Park, Robert H. Hice, Margaret C. Wirth, Brian A. Federici

**Affiliations:** 1 Department of Entomology, University of California, Riverside, Riverside, CA 92521 USA; 2 Department of Biological Sciences, California Baptist University, Riverside, CA 92504 USA; 3 Interdepartmental Graduate Program in Microbiology and Institute for Integrative Genome Biology, University of California, Riverside, Riverside, CA 92521 USA

## Abstract

Two mosquitocidal bacteria, *Bacillus thuringiensis* subsp. *israelensis* (Bti) and *Lysinibacillus sphaericus* (Ls) are the active ingredients of commercial larvicides used widely to control vector mosquitoes. Bti’s efficacy is due to synergistic interactions among four proteins, Cry4Aa, Cry4Ba, Cry11Aa, and Cyt1Aa, whereas Ls’s activity is caused by Bin, a heterodimer consisting of BinA, the toxin, and BinB, a midgut-binding protein. Cyt1Aa is lipophilic and synergizes Bti Cry proteins by increasing midgut binding. We fused Bti’s Cyt1Aa to Ls’s BinA yielding a broad-spectrum chimeric protein highly mosquitocidal to important vector species including *Anopheles gambiae*, *Culex quinquefasciatus*, and *Aedes aegypti*, the latter an important Zika and Dengue virus vector insensitive to Ls Bin. Aside from its vector control potential, our bioassay data, in contrast to numerous other reports, provide strong evidence that BinA does not require conformational interactions with BinB or microvillar membrane lipids to bind to its intracellular target and kill mosquitoes.

## Introduction

Mosquitoes transmit many pathogens that cause debilitating diseases including the viruses that cause Dengue, West Nile, Zika and Yellow Fever, nematodes responsible for River Blindness and filariasis, and protozans causing various malarias. Over half the human population lives in areas where these mosquito-vectored pathogens are endemic, with the principal vectors being species of *Aedes*, *Anopheles*, and *Culex* mosquitoes. Recent data from the World Health Organization show that more than 3 billion people are at risk of malaria alone, with an estimated 214 million cases and greater than 438,000 deaths in 2015, most of the latter being children who die under the age of 5, making malaria the leading cause of morbidity and mortality worldwide^1^. The incidence of Dengue and Yellow Fever is also high, with respectively, 50–100 million and 200,000 cases occurring yearly^2, 3^.

Synthetic chemical insecticides are still used to control mosquitoes. However, their detrimental environmental effects and resistance to these in target populations^4^ led to the development of commercial larvicides based on two mosquitocidal bacteria, *Bacillus thuringiensis* subsp. *israelensis* (Bti) and *Lysinibacillus sphaericus* (Ls). Both produce mosquitocidal protein crystals and have been used widely in mosquito control programs for decades^5–7^. When ingested, these protein crystals dissolve in the alkaline larval midgut, are proteolytically activated, and bind to microvillar receptors forming lesions that destroy midgut cells leading to larval death^5–9^. Cyt1Aa (27.5 kDa) differs from the Bti Cry and Ls proteins in that it does not require a glycoprotein receptor, but rather binds with high affinity to lipids in the microvillar plasmlemma^10^. It has only low toxicity to mosquito larvae, but is important to toxicity in that it synergizes Bti Cry and Ls mosquitoicdal proteins and delays resistance to these^11^. After binding, Cyt1Aa is thought to act by forming pores or lipid faults in the microvillar membrane^10^.

The Ls binary toxin (Bin) is a heterodimer of two related propeptides, BinA, a toxin (42 kDa), and BinB, a midgut microvillar binding protein (51 kDa), which co-crystallize during synthesis^5, 6, 12–14^. BinB binds to a glycoprotein receptor, the first identified being a glycosylphosphatidylinositol (GPI)-anchored α-glucosidase^15^. Most *Aedes* and many *Anopheles* species lack this type of receptor and thus are not sensitive to Bin^12^. Unlike Bti proteins that act at the microvillar surface, BinA and BinB are internalized and act intracellularly killing cells by autophagy and/or apoptosis^5, 16, 17^, during which large cytoplasmic vacuoles are formed followed by midgut exfoliation that results in larval death. Several studies suggest that interaction of BinA and BinB is required for toxicity^18–21^. At LC_90_ levels, Ls mortality peaks at 48 hours post-treatment due to Bin’s internalization process, whereas with Bti maximum mortality occurs at 24 hours post-treatment.

## Results

Previous recombinant Bti strains we constructed containing various combinations of mosquitocidal Cry proteins, Cyt1Aa, and Bin are much more potent than wild type strains of Bti and Bs, and avoid resistance^13, 14^. These recombinants demonstrate bacterial insecticdes can be improved significantly through genetic engineering and synthetic biology techniques, suggesting other novel combinations of high efficacy are possible. In a proof-of-concept study, we fused the Cyt1Aa protoxin, which has high affinity for mosquito microvilli lipids, to the BinA protoxin, yielding the chimeric protein, Cyt1Aa-BinA (69.6 kDa). We then evaluated this construct for stable synthesis in 4Q7, an acrystalliferous strain of Bti, and for the efficacy of this recombinant chimeric strain against larvae of mosquito species belonging to the three most important genera of disease vectors, *Anopheles*, *Aedes*, and *Culex*. Here we show this chimeric strain forms a stable parasporal inclusion in Bti and is highly toxic to *Anopheles gambiae, An. stephensi*, *Aedes aegypti*, and both Bin-sensitive and Bin-resistant strains of *Culex quinquefasciatus*. The high toxicity we obtained against *Ae. aegypti* is potentially important, as our chimera expanded the target spectrum of BinA to include this species, which lacks a BinB receptor and thus is poorly sensitive to Ls Bin^1^.

**Figure 1:**
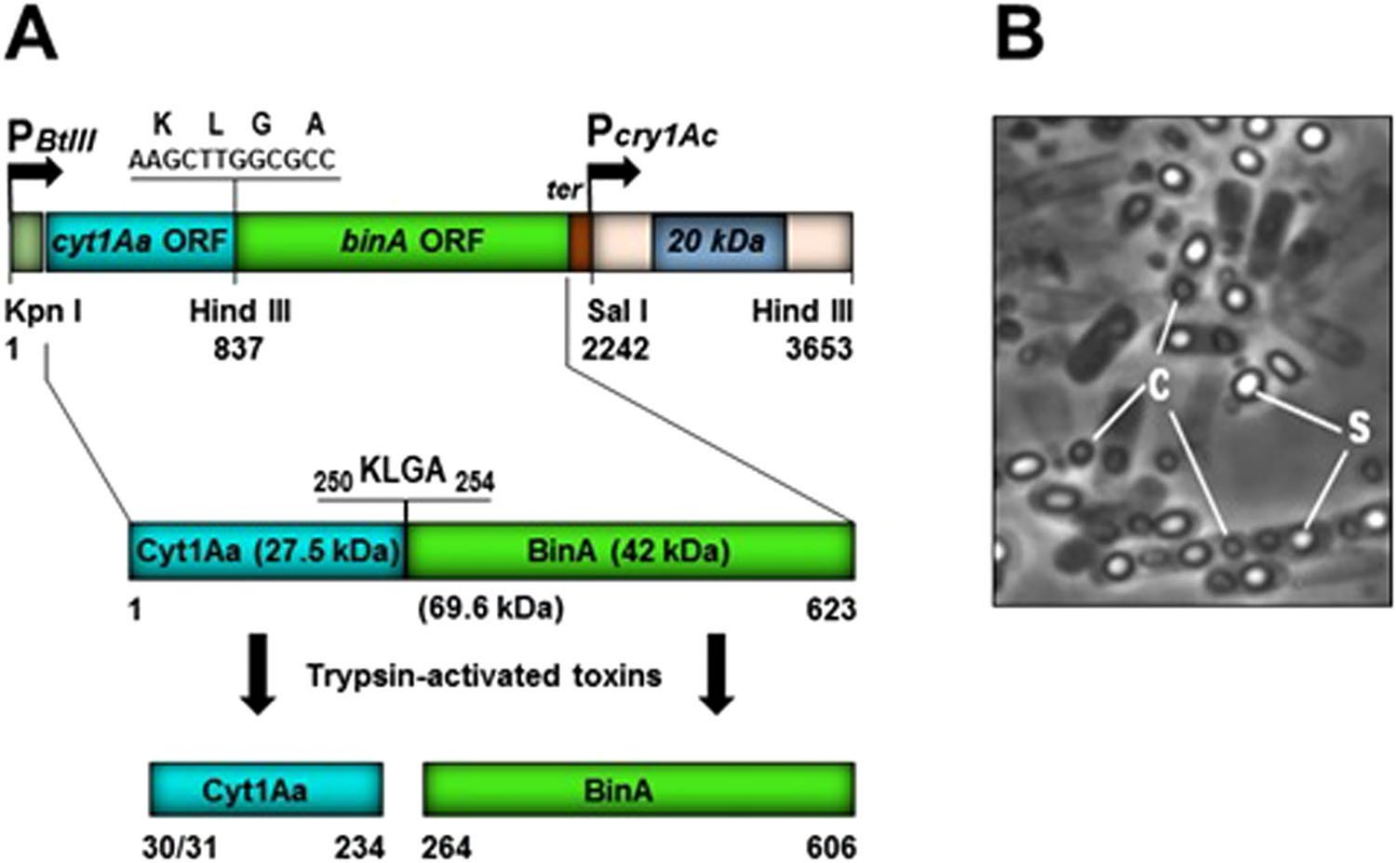
Parasporal inclusions of chimeric Cyt1Aa-BinA synthesized using the 4Q7 acrystalliferous strain of *Bacillus thuringiensis* subsp. *israelensis*. (**A**) Schematic of the *cyt1Aa-binA* gene fusion. A 0.84-kbp fragment containing the *cyt1Aa* gene BtIII promoter (P*BtIII*) and *cyt1Aa* open reading frame (ORF) was cloned in frame with a 1.4 kbp fragment harboring the *binA* ORF flanked by its native transcription terminator (*ter*). The nucleotide sequences at the fusion site (underlined) and the coded amino acids (KLGA, lysine-leucine-glycine-alanine) are shown above the *Hin*dIII site, as are the positions of applicable restriction sites and the *20-kDa* like chaperone-like protein gene under control of the *cry1Ac* gene promoter (P*cry1Ac*) used for cloning in pBU4 to generate the expression vector pBU-*cyt1Aa-binA*. The Cyt1Aa-BinA protoxin is composed of 623 amino acids and has a molecular mass of 69.6 kDa; the predicted proteolytically active forms of Cyt1Aa (22.7 kDa) and BinA (38.8 kDa) are shown. (**B**) Micrograph (x1000) of 4Q7/pBU-*cyt1Aa-binA* grown for 48 hr showing sporulated cells with endospores (s) and parasporal inclusions (c); free spores and inclusions are also present, which is typical after lysis of *B. thuringiensis* cells.

**Figure 2:**
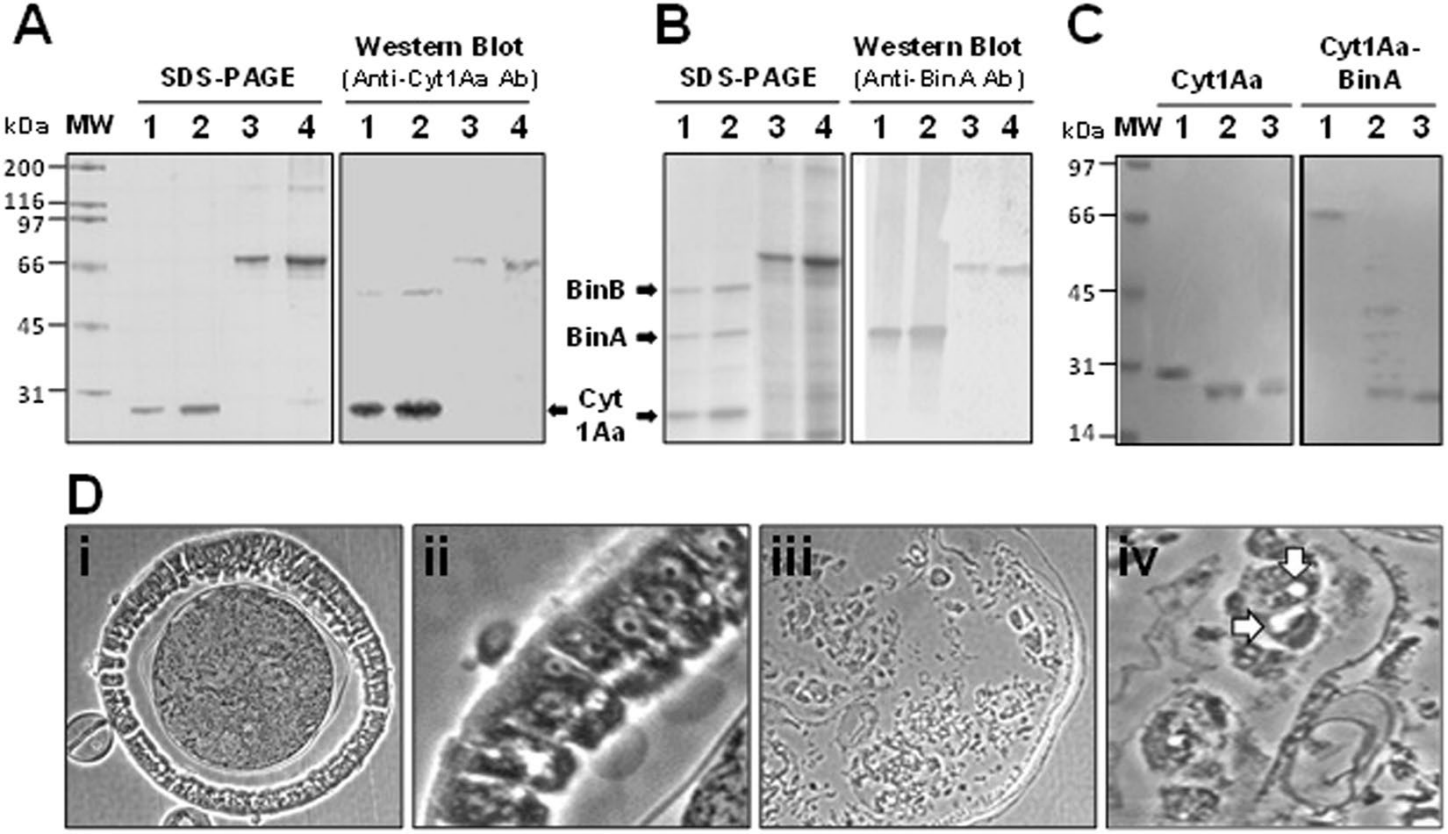
Protein profile and antigenicity of the Cyt1Aa-BinA chimera. Inclusion bodies were purified from *Bacillus thuringiensis* subsp*. israelensis* 4Q7 strains producing (**A**) Cyt1Aa (4Q7/pWF45; lanes 1, 2), Cyt1A-BinA (4Q7/pBU-*cyt1Aa-binA*; lanes 3, 4), (**B**) Cyt1Aa and BinAB (4Q7/45S1; lanes 1, 2) or Cyt1A-BinA (4Q7/pBU-*cyt1A-binA*; lanes 3, 4). Inclusions were solubilized and fractionated by SDS-PAGE in a 10% gel and electroblotted for Western analysis using rabbit anti-Cyt1Aa and anti-BinA antibodies (Ab). Lanes 1, 2, and 3, 4, respectively, 0.75 μg and 1.5 μg of protein; molecular masses: Cyt1Aa, 27.2 kDa; BinA, 42 kDa; BinB, 52 kDa; and Cyt1Aa-BinA 69.6 kDa. **(C)** SDS-PAGE demonstrating proteolytic cleavage of Cyt1Aa-BinA by trypsin, with Cyt1Aa as a control. Purified parasporal inclusions were solubilized in 50 mM NaOH, supernatants collected and neutralized with HCl, and digested with the enzyme at 28 °C. Untreated samples, 1.5 hr (lane 1), and trypsin-treated samples, 0.5 hr (lane 2) and 1.5 hr (lane 3). (**D**) Midgut histopathology caused by Cyt1Aa-BinA chimera in fourth instars of *Culex quinquefasciatus* 8 hours post-treatment at the LC_95_ concentration; Control midgut epithelium, (i) and (ii), respectively, 100x and 400x magnification. Midgut epithelium of a treated larva (iii) and (iv), respectively 100x and 600x magnification. Note the vacuoles in cells designated by arrows in **D** (iv) that have sloughed from the midgut basement membrane (**C**, 100x; **D**, 600x). The central circular area in A is the food column surrounded by the peritrophic membrane. MW, protein molecular mass standards; kDa, kilodaltons.

The Bti 4Q7 strain that synthesized Cyt1Aa-BinA (Fig. 1A) produced spores and parasporal bodies within 24–36 hr of incubation in NBG broth or on Nutrient agar (Fig. 1B). The parasporal bodies were released from fully lysed cells, or remained associated with the spore. The chimeric strain kept in NBG broth or Nutrient agar was stable for at least six months at 4 °C, as determined by microscopy and SDS-PAGE. To show parasporal bodies contained the Cyt1Aa-BinA chimera, they were separated from spores on a sucrose gradient and analyzed by SDS-PAGE and Western blot analyses. A single protein of ~70 kDa, the predicted mass of Cyt1Aa-BinA, was observed, and this protein reacted with anti-Cyt1Aa and anti-BinA antibodies (respectively, Fig. 2A and B). When subjected to digestion with trypsin, the Cyt1Aa-BinA chimera yielded fragments consistent with normal cleavage products of Cyt1A and BinA (Fig. 2C).

Against all larvae, bioassays using the Cyt1Aa strain showed negligible toxicity, with LC_50_s ranging from 4,219–47,370 ng/ml and LC_95_s from 13,722–155,050 ng/ml (Table 1). Bti 4Q5, a strain that produces its four major toxins (Cry4Aa, Cry4Ba, Cry11Aa and Cyt1Aa), was the most potent (LC_50s_ 3.6–7.1 ng/ml, LC_95s_ from 18.5–88 ng/ml). Ls 2362 was active against *Cx. quinquefasciatus*, and *An. gambiae* and *An. stephensi*, but not against *Ae. aegypti* and *Cx. quinquefasciatus* BS-R, a strain selected for high levels of resistance to Bin; the LC_50_s and LC_95_s of Ls 2362 were >1,000,000 ng/ml. The Cyt1Aa-BinA chimeric strain, however, was highly toxic to larvae of species belonging to all three major genera of disease vectors, *Culex, Aedes* and *Anopheles*, with LC_50_s ranging from 9.2 to 61.9 ng/ml, and LC_95_s from 30 to 271 ng/ml (Table 1). Toxicity of the chimera was high by 24 hours post-treatment (Table 1), which typically only occurs by 48 hours when Ls is tested against larvae (Table 2).

**Table 1:** Toxicity of the Cyt1Aa-BinA chimera and control strains of *Bacillus thuringiensis* subsp. *israelensis* (Bti) and *Lysinibacillus sphaericus* (Ls) to 4^th^-instars of *Aedes*, *Anopheles* and *Culex* species at 24 hours post-treatment^a^.

Bacterial Strain (Toxins produced)	LC_50_ (Fiducial Limits)	RT^d^-LC_50_	LC_95_ (Fiducial Limits)	RT^d^-LC_95_	Slope
*Culex quinquefasciatus* S-Lab					
Bti 4Q7/pWF45 (Cyt1Aa)	47,370.1 (35,629.5–59,705.4)	6,671	155,050.3 (114,040.8–260,168.4)	1,914.2	3.2 ± 0.5
Ls 2362 (BinAB)	181.4 (118.1–296.3)	25.6	7,569.4 (3,009.6–33,192.5)	93.5	1.0 ± 0.1
Bti 4Q7/*cyt1Aa-binA* (Cyt1Aa-BinA chimera)	9.2 (7.2–12.1)	1.3	30.5 (20.2–71.2)	0.4	3.2 ± 0.6
Bti 4Q5 (Cry4A, Cry4B, Cry11A, Cyt1A)	7.1 (2.6–18.6)	1.0	81.0 (16.7–427.3)	1.0	1.6 ± 0.4
*Culex quinquefasciatus* BS-R^b^					
Bti 4Q7/pWF45 (Cyt1Aa)	27,022.4 (20,523.2–35,262.4)	5,630	100,175.2 (70,685.6–169,448.0)	4,595.2	2.9 ± 0.4
Ls 2362 (BinAB)	>1,000,000^c^	>208,333	>1,000,000^c^	>45,872	—
Bti 4Q7/*cyt1Aa-binA* (Cyt1Aa-BinA chimera)	10.2 (2.6–41.4)	2.1	42.0 (2.2–1,214.9)	1.9	2.7 ± 1.0
Bti 4Q5 (Cry4A, Cry4B, Cry11A, Cyt1A)	4.8 (3.5–6.3)	1.0	21.8 (14.4–45.3)	1.0	2.5 ± 0.4
*Aedes aegypti*					
Bti 4Q7/pWF45 (Cyt1Aa)	4,218.7 (2,997.1–5,709.8)	1,172	22,764.9 (14,634.9–48,334.1)	84	2.3 ± 0.4
Ls 2362 (BinAB)	>1,000,000	>277,778	>1,000,000 ^c^	>3,689	—
Bti 4Q7/*cyt1Aa-binA* (Cyt1Aa-BinA chimera)	61.9 (46.7–80.1)	17.2	271.1 (185.1–513.4)	14.7	2.6 ± 0.4
Bti 4Q5 (Cry4A, Cry4B, Cry11A, Cyt1A)	3.6 (2.5–4.8)	1.0	18.5 (11.9–39.6)	1.0	2.3 ± 0.4
*Anopheles gambiae*					
Bti 4Q7/pWF45 (Cyt1Aa)	46,557.3 (13,980.9–137,613.6)	1,757	129,978.8 (25,520.6–923,387.1)	1,465.4	3.7 ± 1.4
Ls 2362 (BinAB)	201.2 (154.6–260.4)	7.6	1,150.9 (760.7–2,206.5)	13	2.2 ± 0.3
Bti 4Q7/*cyt1Aa-binA* (Cyt1Aa-BinA chimera)	23.0 (17.6–30.0)	0.9	80.9 (57.4–135.4)	0.9	3.0 ± 0.4
Bti 4Q5 (Cry4A, Cry4B, Cry11A, Cyt1A)	26.5 (20.2–34.3)	1.0	88.7 (63.9–144.8)	1.0	3.1 ± 0.4
*Anopheles stephensi*					
Bti 4Q7/pWF45 (Cyt1Aa)	7,779.5 (6,758.9–9,028.8)	526	13,722.2 (11,212.0–20,809.5)	195	6.7 ± 1.3
Ls 2362 (BinAB)	707.5 (620.3–808.5)	48	1,179.2 (992.0–1,618.5)	17	7.4 ± 1.3
Bti 4Q7/*cyt1Aa-binA* (Cyt1Aa-BinA chimera)	28.9 (22.0–37.2)	1.9	93.1 (67.8–149.8)	1.3	3.2 ± 0.4
Bti 4Q5 (Cry4A, Cry4B, Cry11A, Cyt1A)	14.8 (11.1–19.8)	1.0	70.4 (46.8–133.2)	1.0	2.4 ± 0.3

^a^24 hr-mortality, ng/ml. ^b^Laboratory strain selected for high-level resistance to the binary toxin (Bin) of Ls 2362. ^c^No mortality at 1 mg/ml. ^d^Relative toxic values of recombinant Bti strains and Ls 2362 against mosquito larvae when compared to strain Bti 4Q5.

**Table 2:** Toxicity of the Cyt1Aa-BinA chimeric strain of *Bacillus thuringiensis* subsp. *israelensis* 4Q7/Cyt1Aa-BinA or *Lysinibacillus sphaericus* (Ls) wild type strain to 4^th^-instars of *Culex quinquefasciatus* at 24 versus 48 hours post treatment.

Bacterial Strain	LC_50_ (Fiducial Limits)^a^	LC_95_ (Fiducial Limits)^a^	Slope
24 hours			
Ls 2362 (BinAB)	218.7 (158.5–308.0)	2,548.5 (1,396.8–6,667.3)	1.5 ± 0.2
Bti 4Q7/ *cyt1Aa-binA* (Cyt1Aa-BinA chimera)	6.5 (3.3–13.0)	38.5 (11.0–174.6)	2.1 ± 0.5
48 hours			
Ls 2362 (BinAB)	19.5 (14.7–25.9)	90.5 (61.5–160.3)	2.5 ± 0.3
Bti 4Q7/ *cyt1Aa-binA* (Cyt1Aa-BinA chimera)	5.8 (2.7–12.5)	31.0 (7.6–151.8)	2.3 ± 0.6

^a^ng/ml.

Interestingly, with regard to both LC_50_s and LC_95_s, the relative toxicities of the Cyt1Aa-BinA chimera or Bti 4Q5 (with the wild type parasporal body) against all larvae assayed, with the exception of *Ae. aegypti*, were not significantly different, as they ranged from 0.4–2.1, even against the BinA/BinB-resistant *Cx. quinquefasciatus* BS-R strain (Table 1). Against the anopheline species, although fiducial limits of LC_50_s of the Cyt1Aa-BinA protein (23.0 ng/ml) and Bti 4Q5 (26.5 ng/ml) against *An. gambiae* overlapped, those of Cyt1Aa-BinA (28.9 ng/ml) and Bti 4Q5 (14.8 ng/ml) against *An. stephensi* did not. However, their LC_95_s completely overlapped against both species indicating that the Cyt1Aa-BinA fusion protein alone was as effective as the wild-type Bti 4Q5.

Perhaps most interesting are the LC_50_s and LC_95_s toxicities observed for Cyt1Aa-BinA against *Ae. aegypti*, respectively, 61.9 ng/ml and 271.1 ng/ml, when compared to Ls (>1,000,000 ng/ml), i.e., the chimera was >16,155 and >3689 more toxic than Ls.

Preliminary histological studies of treated versus control larvae showed that the midgut epithelium was completely destroyed in moribund and dead larvae by eight hours post-treatment at the LC_95_ level (Fig. 2D). Most midgut cells had sloughed from the basement membrane and had lysed. Those that still had a recognizable cellular structure lacked microvilli and had one or two large vacuoles in the cytoplasm, the chacteristic cytopathology resulting from Ls Bin intoxication.

In the present study we fused the protoxins, not the activated toxins, so that the protoxin chimera contained proteolytic cleavage sites of each partner. Once activated in the midgut lumen each partner should then act independently, Cyt1Aa causing midgut microvillar membrane lesions through which BinA would enter the cytoplasm to reach its internal target site, killing the cell within 24 hr rather than the 48 hours required by the BinAB complex^5, 22, 23^. Our trypsin activation and Western blot results (Fig. 2C) indicate the two partners separated and acted independently, achieving toxicity within 24 hr for all mosquito species and strains tested (Table 1) as opposed to 48 hr with wild type BinAB. Our purpose did not include determining the type of Cyt1Aa lesion formed. However, with a diameter of about 3 nm, BinA is too large^6^ to be a cation ion channel (1–2 nm)^9, 24^, and more likely forms an irregular lipid fault^25^ as opposed to a larger semicircular pore. In fact, in a previous study^7^ we showed that activated the BinAB complex, about 6 nm in diameter^6^, can enter *Cx. quinquefasciatus* midgut cells resistant to Bin *in vivo* through Cyt1Aa lesions without binding to microvilli.

## Discussion

High toxicity to *Ae. aegypti* (Table 1) was unexpected because this species does not have a Bin receptor and Cyt1Aa’s effect is negligible. However, we previously showed combination of Ls technical powder with purified Cyt1Aa crystals at a 10:1 ratio increased toxicity slightly to *Ae. aegypti*, with LC_50_ and LC_95_ values of 3,800 ng/ml and 31,500 ng/ml, and synergism factors of 2.1 and 8.6, respectively^26^. Compared with these results, LC_50_ and LC_95_ values for the chimera (a 1:1 ratio of Cyt1Aa:BinA) increased toxicity to this species by, respectively, 55-fold and 116.2-fold. The marked differences in LC_50_ and LC_95_ values between these two studies cannot be compared directly due to variations in toxin constructs, but our chimera’s high toxicity to four mosquito species indicates that the intracellular target for BinA is present in *Ae. aegypti*, and thus probably in all mosquito species. Moreover, against *An. gambiae*, it is the most toxic of any strain we tested (Table 1). Based on our SDS-PAGE results we estimate BinA is only about 10% (dry weight) of spore/parasporal body complex tested, indicating its activated peptide is one of the most potent mosquitocidal toxins known, if not the most toxic. Although not as toxic to *Ae. aegypti* as Bti 4Q5 with the wild type parasporal body (LC_50_ = 3.6 ng/ml, LC_95_ = 18.5 ng/ml), these results demonstrate that the Cyt1Aa-BinA chimera strain extended the target spectrum of Ls BinA (Table 1). Thus, rather than using a mixture of Bti and Ls, as is currently done is some current commercial products, the Cyt1Aa-BinA chimera combines the properties of high toxicity against a broad vector target spectrum with the known resistance management properties of Cyt1A^11^.

Aside from potential vector control applications, the Cyt1Aa-BinA chimera could prove useful for clarifying how BinA kills midgut cells causing mosquito death. The literature on these topics is full of disparate and often contradictory results. Whereas Bin’s intoxication has been well described cytologically^5, 16–18^, its mode of action at the molecular level remains unknown. In many studies over the past decade it appears to be assumed that BinA and BinB cystallize separately in Ls, dissolve after ingestion in the midgut, are activated, and then associate to form an activated dimer or tetramer^20–22, 27^ (2BinA + 2BinB; see Fig. 6)^20^. However, strong evidence for initial independent dissociation or tetramer formation is lacking in any of these studies. In earlier studies^13, 14^ it was shown expression of the *bin* operon, i.e., *binA* and *binB*, yielded only a single crystal, demonstrating BinA and BinB formed a heterodimer, not separate crystals, which was confirmed recently by the the solution of Bin’s crystal structure^6^. Another problem with a report that BinA and BinB prepared separately and then mixed together formed a tetramer^28^ is that in a subsequent study it was shown the Ls protein complex studied^29^ by the former group was a spore coat protein complex, not the Bin toxin. In other studies it has been suggested reassociation of BinA and BinB may be required for important conformational changes essential to both molecules so that activated Bin can bind receptors, interact with membrane lipids for additional structural alterations, and induce its internalization^18, 20, 27^. We do not question these results under the conditions tested, but our *in vivo* results reported here provide strong evidence that BinA once activated is highly toxic without requiring BinB for conformational changes, nor does it appear to require interactions with microvillar membrane lipids for toxicity. This suggests that BinA’s hydrophobic domains may target this toxin to an intracellular organelle, such as the endoplasmic reticulum, rather than act by forming pores in the microvillar membrane.

## Materials and Methods

### Bacterial strains, culture media, and DNA extraction

The DH5α strain of *Escherichia coli* (Invitrogen) was used for cloning and amplifying plasmid DNA. The strains of crystalliferous *B*. *thuringiensis* subsp. *israelensis* (Bti) 4Q5, acrystalliferous Bti 4Q7, and *L. sphaericus* (Ls) 2363 were obtained from the *Bacillus* Genetic Stock Center (Ohio State University, Columbus, OH). Erythromycin-resistant recombinants 4Q7/pWF45 and 4Q7/p45S1, producing, respectively, Cyt1Aa and BinA/BinB (42 kDa/51 kDa) parasporal bodies have been described previously^13, 30–32^. All strains were maintained on Nutrient agar (Becton Dickinson, Sparks, MD) throughout the study. LB medium (Becton Dickinson, Sparks, MD) was used for growing *E. coli* and extracting plasmid DNA using the Wizard Plus Mini-prep DNA Purification system (Promega). Genomic DNA was extracted using the DNeasy Blood and Tissue Kit (Qiagen).

### Construction of pBU-*cyt1Aa-binA*

To make a construct that synthesizes the Cyt1Aa-BinA chimera, plasmid pWF53^30^ was digested with *Sal*I and *Hin*dIII (FastDigest, Thermo Scientific) and the 1.4-kb fragment that contains the *cry1Ac* promoter controlling expression of the *20-kDa* chaperone-like gene was ligated into plasmid pBU4^31^ digested with the same enzymes and treated with FastAP alkaline phosphatase (Thermo Scientific) to generate plasmid pBU-*Pcry1Ac-20kDa* (8.8 kbp). A 0.84-kb fragment containing the *cyt1Aa* BtIII promoter and the *cyt1Aa* open reading frame (ORF) (GenBank AL7317825) was obtained by PCR using the Phire Hot Start II polymerase (Thermo Scientific), primer pair CytF: 5′-gggtaccATTTGATAATAATTGCAAGTTTAAAATCAT-3′ and Cyt1R 5′-gggcgccaagcttGAGGGTTCCATTAATAGCGCTAGTAAGATCTG-3′ and 4Q5 genomic DNA preparation which contained template pBtoxis (GenBank NC_010076). The amplicon was digested with *Kpn*I and *Hin*dIII. Similarly, a 1.4-kbp PCR amplicon containing the *binA* ORF and Bin transcription terminator (GenBank M20390) was obtained by PCR using Ls 2362 genomic DNA and the primer pair DB42F 5′-aaagcttggcgccATGAGAAATTTGGATTTTATTGATTC-3′ and DB42R 5′-ggtcgacAAACAACAACAGTTTACATTCGAGTG-3. The amplicon was digested with *Hin*dIII and *Sal*I. To generate pBU-*cyt1Aa-binA* (Fig. 1A), pBU-*20kDa* was digested with *Kpn*I and *Sal*I, treated with FastAP (Thermo Scientific), and ligated to the 0.84 kbp *Kpn*I/*Hin*dIII and 1.4 kbp *Hin*dIII/*Sal*I digested fragments.

### Transformation

Bti 4Q7 was transformed by electroporation as previously described^13, 14^, and transformants (4Q7/pBU-*cyt1Aa-binA*) were selected on LB agar with tetracycline (3 μg/ml) at 28 °C.

### Bacterial strains and purification of parasporal bodies

Ls 2362 was grown in MBS broth^25^, and Bti strains 4Q5, 4Q7/pWF45, 4Q7/pBU-*cyt1Aa-binA*, and 4Q7/p45S1 were grown in 50 ml of NBG^13, 14^ appropriately supplemented with 25 μg/ml erythromycin and 3 μg/ml tetracycline, at 28 °C for 4 days by which time >95% of the cells had sporulated and lysed. Spores and crystals were collected by centrifugation at 6,500 *g* for 15 min, washed 2x in double-distilled (dd) H_2_O, followed by centrifugation at 6,500 *g* for 15 min at 4 °C after each wash, and lyophilized (FreezeZone 4.5, Labconco) for storage.

To isolate parasporal bodies, spore/parasporal body mixtures collected from 50 ml cultures were resuspended in 15 ml ddH_2_O and sonicated twice at 50% duty cycle for 15 s using the Ultrasonic Homogenizer 4710 (Cole-Parmer Instrument Co.). Five-milliliter samples were loaded onto a sucrose gradient cushion (30–65% w/v), which was then centrifuged at 20,000 *g* for 45 min at 20 °C in a Beckman L7–55 ultracentrifuge using the SW28 rotor. Bands containing parasporal bodies were collected and washed twice in ddH_2_O, followed by centrifugation at 6,500 *g* for 15 min at 4 °C after each wash and lyophilized for storage.

### Western blot analysis

Purified parasproal bodies (~10 μg) were solubilized in alkaline buffer (50 mM Na_2_CO_3_, pH 11) and protein concentration was determined by the method of Bradford, as described previously^13, 14^. Protein samples (0.75 μg and 1.5 μg) were fractionated by electrophoresis in an SDS–10% polyacrylamide gel and electroblotted onto a polyvinylidene difluoride membrane (MicronSeparations, Inc.) using a model PS50 electroblotter (Hoefer Scientific Instruments). Western blot analysis was performed using primary rabbit anti-BinA and anti-Cyt1Aa antibodies and alkaline phosphatase-conjugated goat anti-rabbit immunoglobulin G (Southern Biotechnology Associates, Inc., Birmingham, AL) as the secondary antibody. Binding of the secondary antibody was detected with the nitroblue tetrazolium and 5-bromo-1-chloro-3-indolyl phosphate (BCIP) reagents (Promega).

### Trypsin digest

Approximately 5 μg of purified parasporal bodies were solubilized in 25 μl 50 mM NaOH, 25 °C for 10 min, followed by addition of 25 μl of 50 mM HCl. Samples were spun at 16,000 g for 5 min to remove the insoluble fraction, and supernatants were collected and activated with 1:50 (w/w) trypsin (Sigma) for 2 h at 25 °C. The products liberated by proteolytic cleavage were analyzed by SDS-PAGE as previouslty described^13, 14^.

### Microscopy

Sporulating cultures were monitored and photographed with a DMRE phase-contrast microscope (Leica) at a magnification of 1,000x. For preliminary histological studies, control, moribund, and dead larvae (LC_95_ level) were fixed, dehydrated, and embedded in Epon-Araldite^14^. Sections 0.25–0.50 μm thick were cut and examined with the above phase contrast microscope.

### Bioassays

Lyophilized cultures containing spores and parasporal bodies of the Bti and Ls strains were resuspended in ddH_2_O. Suspensions were diluted to 6 to 7 different concentrations, ranging from 0.5 ng/ml to 1 µg/ml, in 6 oz cups in a final volume of 100 ml. Bioassays were replicated three times using 30 fourth-instars of S-Lab (Bin-sensitive) and BS-R (Bin-resistant) strains of *Cx. quinquefasciatus*, *Ae. aegypti, An. gambiae* (courtesy of B. J. White, Department of Entomology, University of California, Riverside, CA) and *An. stephensi* (courtesy of A. A. James, Department of Molecular Biology and Biochemistry, University of California, Irvine) per concentration. After 24 h of exposure at 28 °C, dead larvae were counted and the 50% and 95% lethal concentrations, respectively, LC_50_ and LC_95_, were calculated by Probit analysis (POLO-PC; LeOra Software, Berkeley, CA)^26^.

### Availability of data

All reagents and data described in this manuscript are available upon request.

## References

[1] WHO Factsheet on the World Malaria report, December. http://www.who.int/malaria/media/world-malaria-report-2015/en/http://www.who.int/malaria/media/world_malaria_report_2014/en/ (2015).

[2] Huang YJ, Higgs S, Horne KM, Vanlandingham DL (2014). Flavivirus-Mosquito Interactions. Viruses.

[3] Mathai D, Vasanthan AG (2009). State of the Globe: Yellow Fever is Still Around and Active. J. Glob. Infect. Dis..

[4] Enayati A, Hemingway J (2010). Malaria management: past, present, and future. Ann. Rev. Entomol..

[5] Berry C (2012). The bacterium, *Lysinibacillus sphaericus*, as an insect pathogen. J. Invertebr. Pathol..

[6] Colletier J-P (2016). *De novo* phasing with X-ray laser reveals mosquito larvicide BinAB structure. Nature.

[7] Federici BA, Park H-W, Bideshi DK, Wirth MC, Johnson JJ (2003). Recomninant bacteria for mosquito control. J. Exp. Biol..

[8] Ben-Dov E (2014). *Bacillus thuringiensis* subsp. *israelensis* and its dipteran-specific toxins. Toxins.

[9] Promdonkoy. B, Ellar DJ (2000). Membrane pore architecture of a cytolytic toxin from *Bacillus thuringiensis*. Biochem J..

[10] Butko P (2003). (2003) Cytolytic toxin Cyt1A and its mechanism of membrane damage: data and hypothesis. Appl. Environ. Microbiol..

[11] Wirth MC (2010). Mosquito resistance to bacterial larvicidal toxins. Open Toxinol. J..

[12] Nicolas L, Nielsen-LeRoux C, Charles J-F, Delecluse A (1993). Respective roles of the 42- and 51-kDa components of the *Bacillus sphaericus* toxin overexpressed in. Bacillus thuringiensis. FEMS Microbiol. Lett..

[13] Park H-W, Bideshi DK, Federici BA (2003). Recombinant strain of *Bacillus thuringiensis* producing Cyt1A, Cry11B, and the *Bacillus sphaericus* binary toxin. Appl. Environ. Microbiol..

[14] Park H-W (2005). Recombinant larvicidal bacteria with markedly improved efficacy against *Culex* vectors of West Nile virus. Am. J. Trop. Med. Hyg..

[15] Darboux I, Nielsen-LeRoux C, Charles J-F, Pauron D (2001). The receptor of *Bacillus sphaericus* binary toxin in *Culex pipiens* (Diptera: Culicidae) midgut: molecular cloning and expression. Insect Biochem Mol Biol.

[16] Opota O (2011). *Bacillus sphaericus* binary toxin elicits host cell autophagy as a response to intoxication. PLoS One.

[17] Tangsongcharoen C, Chomanee N, Promdonkoy B, Boonserm P (2015). *Lysinibacillus sphaericus* binary toxin induces apoptosis in susceptible *Culex quinquefasciatus* larvae. J. Invertebr. Pathol..

[18] Boonserm P (2006). Association of the components of the binary toxin from *Bacillus sphaericus* in solution and with model lipid bilayers. Biochem. Biophys. Res. Comm..

[19] Limpanawat S, Promdonkoy B, Boonserm P (2009). The C-terminal domain of BinA is responsible for *Bacillus sphaericus* binary toxin BinA-BinB interaction. Curr. Microbiol..

[20] Srisucharitpanit K (2014). Crystal structure of BinB: A receptor binding component of the binary toxin from *Lysinibacillus sphaericus*. Proteins.

[21] Kale A, Hire RS, Hadapad AB, D’Souza SF, Kumar V (2013). Interaction between mosquito-larvicidal *Lysinibacillus sphaericus* binary toxin components: Analysis of complex formation. Insect Biochem. Mol. Biol..

[22] Lekakarn H, Promdonkoy B, Boonserm P (2015). 2015. Interaction of *Lysinibacillus sphaericus* binary toxin with mosquito larval gut cells: Binding and internalization. J Invertebr Pathol.

[23] Clark MA, Baumann P (1990). Deletion analysis of the 51-kilodalton protein of the *Bacillus sphaericus* 2362 binary mosquitocidal toxin: Construction of derivatives equivalent to the larva-processed toxin. J. Bacteriol.

[24] Cantón PE, López-Díaz JA, Gill SS, Bravo A, Soberón S (2014). Membrane binding and oligomer membrane insertion are necessary but insufficient for *Bacillus thuringiensis* Cyt1Aa toxicity. Peptides.

[25] Manceva SD, Pusztai-Carey M, Russo PS, Butko P (2005). A detergent-like mechanism of action of the cytolytic toxin Cyt1A from *Bacillus thuringiensis* var. israelensis. Biochem..

[26] Wirth MC, Federici BA, Walton WE (2000). Cyt1A from *Bacillus thuringiensis* synergizes activity of *Bacillus sphaericus* against *Aedes aegypti* (Diptera: Culicidae). Appl. Environ. Microbiol..

[27] Surya W, Chooduang D, Choong YK, Torres J, Boonserm P (2016). (2016). Binary toxin subunits of *Lysinibacillus sphaericus* are monomeric and form heterodimers after *in vitro* activation. PLoS ONE.

[28] Smith AW, Camara-Artigas A, Brune DC, Allen JP (2005). Implications of high-molecular weight oligomers of the binary toxin from *Bacillus sphaericus*. J. Invertebr. Pathol..

[29] Hire RM, Sharma M, Hadapad AB, Kumar V (2014). (2014) An oligomeric complex of BinA/BinB is not formed *in-situ* in mosquito-larvicidal *Lysinibacillus sphaericus* ISPC-8. J Invertebr. Pathol..

[30] Wu D, Federici BA (1993). A 20-kilodalton protein preserves cell viability and promotes CytA crystal formation during sporulation in *Bacillus thuringiensis*. J. Bacteriol..

[31] Wu D, Federici BA (1995). Improved production of the insecticidal CryIVD protein in *Bacillus thuringiensis* using *cryIA(c)* promoters to express the gene for an associated 20-kDa protein. Appl. Micriobiol. Biotech..

[32] Bourgouin. C, Delecluse A, de la Torre F, Szulmajster J (1990). Transfer of the toxin protein genes of *Bacillus sphaericus* into *Bacillus thuringiensis* subsp*. israelensis* and their expression. Appl. Environ. Microbiol..

